# Scientific Intolerance

**Published:** 1886-09

**Authors:** 


SCIENTIFIC INTOLERANCE.

How can there be such a question as Science versus Religion? Both are supposed to be expressions of great truths, and though we may not at first sight recognize the harmony of separate presentations, yet if they be not falsities, each is compatible the one with the other. But if any man attempts an appeal to bigotry and prejudice when a new scientific theory is broached, and endeavors to forestall a candid examination by denouncing it as not in harmony with any formulated creed, whether of revealed religion or practical knowledge, he is reviving the methods of the old inquisition, which gave to Galileo the choice between recantation and the stake. Creeds are but the work of men, and in the light of further knowledge even the best may be found defective.

Dr. C. N. Pierce, in the July number of *The Cosmos,* administers a righteous rebuke to the captious cavilers who endeavored to wrest from its obvious sense the meaning of his paper read before the New York Odontological Society last November, and to carry it into the domain of theology. It is always easier to create a prejudice against an argument by branding it as heterodox, than carefully to examine into its merits or demerits. But in the name of all that is consistent, why should political or religious questions enter into the consideration of strictly scientific subjects? If the theories be true they cannot but harmonize with religious truth, and if they be false that must appear upon an examination. The aim of professional societies is supposed to be the elimination of extraneous matter, and the bringing to light of scientific facts, no matter what particular creed or pathy or faction may fancy itself prejudiced thereby. What matters it what political party or religious sect a man may belong to if he reveals to us new teachings of science? Is there not even one place where men of all creeds can meet on common ground, and intelligently discuss scientific and professional questions without engaging in personal, political, or religious polemics? This branding of men as dangerous creatures because they do not subscribe to some other man's dogma, is unworthy those who are searching after truth. What man possesses a creed which all other classes of men will pronounce orthodox? It is every man's privilege to stand up stoutly for his political or religious belief when it is attacked, but it is despicable to appeal to sectarian or party prejudice to prevent the consideration of scientific theories in a professional meeting.

